# Molecular and Other Novel Advances in Treatment of Metastatic Epithelial and Medullary Thyroid Cancers

**DOI:** 10.1155/2010/398564

**Published:** 2010-08-24

**Authors:** David Tai, Donald Poon

**Affiliations:** ^1^Department of Medical Oncology, National Cancer Centre, Singapore 169610; ^2^Duke-NUS Graduate Medical School, 8 College Road, Singapore 169857

## Abstract

An understanding of the mutations of the proto-oncogenes and tumor suppressor genes that occur in thyroid cancers should eventually explain the diverse clinical characteristics of these tumors and also direct therapy. Some insights have already emerged in the last decade; some abnormalities in tumor genes are consistently associated with specific clinical and pathologic findings. These genetic abnormalities usually represent somatic mutations in tumors of follicular epithelial origin, as opposed to inherited mutations in medullary thyroid cancers of parafollicular C cells origin because most thyroid tumors are sporadic and not familial. This is different from the multiple endocrine neoplasia syndromes in which the primary tumorigenic gene mutations are inherited. This improved understanding of the molecular basis of these diseases has led to the development of novel targeted therapeutic approaches which will be discussed in this paper.

## 1. Introduction

Thyroid cancer is the most common form of malignant endocrine tumor. In 2002, the estimated incidence of thyroid cancer worldwide was reported to be in excess of 141 000 [1] with a female predilection (more than 75% being women). The thyroid gland is composed of two main parenchymal cell types. Differentiated thyroid cancers arise from follicular cells giving rise to 3 separate subgroups, papillary, follicular, and hurtle cell. The 3 different subtypes have different clinical behavior. Papillary thyroid cancer (PTC) forming 80% of all thyroid cancers has a 10-year survival rate of more than 90%. PTC displays a spectrum of differentiation from well differentiated, tall cell variant, to poorly differentiated. Follicular thyroid cancer (FTC) constitutes 10% of all thyroid cancers with a wider range of 10-year survival between 43%–94% indicating great heterogeneity within the same disease entity [2–4]. Hurthle cell carcinoma is the most infrequent differentiated carcinoma arising from the follicular cells with incidence estimated to be between 3%–10%. The overall survival rates for Hurthle cell carcinoma are similar or worse compared to follicular carcinoma. One study showed disease-free survival of 40.5% at 10 years [5].

Anaplastic thyroid cancers (ATCs) are undifferentiated tumors of the thyroid follicular epithelium. In marked contrast to differentiated thyroid cancers, anaplastic cancers are extremely aggressive, with a disease-specific mortality approaching 100%. Fortunately, anaplastic thyroid cancer accounts for only 2%–5% of all thyroid cancers [2–4, 6]. Medullary thyroid carcinoma (MTC) is a neuroendocrine tumor which arises from parafollicular or C cells of the thyroid gland. MTC accounts for 3%–5% of all thyroid cancers and portends a higher mortality rate [2, 3].

The prognosis of thyroid cancer as a whole is generally favourable representing only 0.5% (35 000) of all cancer deaths [7]. However, prognosis for patients with metastatic, iodine nonavid, differentiated thyroid cancer and anaplastic cancer remains poor. This paper reviews the therapeutic options available for this subset of patients. Better understanding of molecular pathogenesis of thyroid cancer coupled with significant advances made in cancer therapy guarantees promising and exciting therapeutic options in years to come.

## 2. Differentiated Thyroid Cancer (DTC)

Papillary and follicular thyroid cancers are often treated similarly albeit numerous biologic differences. Standard of care of differentiated thyroid cancer consists of total thyroidectomy often followed by radioactive iodine (RAI) when indicated, aiming for remnant ablation and adjuvant therapy. High-risk patients (age >45, male gender, presence of metastatic disease, extra-thyroidal tumour extension, and tumour diameter >4 cm) should also receive thyroid stimulating hormone (TSH) suppression therapy with thyroid hormone [8]. Up to 25%–50% of patient's tumor loses ability to take up iodine. This is attributed to downregulation of sodium iodide (NaI) symproter (NIS). Promoter methylation of genes required for NaI metabolism leads to decreased expression and is linked to increased signaling through the MAPK pathway [9].

Overall survival (OS) rapidly falls once thyroid cancer metastasize, and is no longer amenable to radioiodine therapy making chemotherapy the only option for systemic treatment. Doxorubicin has been recognized as the standard of care since 1970s based on response rate of 30% in a population of 30 patients [10]. This perhaps is an overestimate of the efficacy of doxorubicin as subsequent trials did not achieve similar response rates. A phase two trial evaluating the efficacy of doxorubicin combined with cisplatin yielded a response rate of less than 10%. The progression-free survival (PFS) time of the patients was estimated at 2 months, and median overall survival was 8 months [11]. Clearly, novel strategies are needed in view of limitations of conventional cytotoxic therapy. Increasing knowledge on cancer thyroid biology has provided an impetus to identifying novel therapeutics and rational trial designs.

Thyroid cancers are highly vascular and express high levels of vascular endothelial growth factor (VEGF) [12]. Inhibition of VEGF receptor signaling alone or in combination has been shown to inhibit growth of thyroid tumors in orthotopic nude mouse [13]. VEGFR therefore seems an attractive and plausible target in iodine refractory metastatic thyroid cancer in humans.

PTC frequently carries gene mutations and rearrangements that lead to activation of the mitogen activated protein kinase (MAPK). Rearrangements of RET and NTRK1 tyrosine kinases, activating mutations of BRAF and RAS are sequential components leading to activation of MAPK. These gene alterations are mutually exclusive given any PTC [14, 15]. PTC is associated with rearrangements of two different transmembrane tyrosine kinase genes: RET and NTRK1. These rearrangements result in the production of chimeric proteins with tyrosine kinase activity that contributes to the development of the malignant phenotype. The chimeric genes resulting from RET rearrangements are referred to as RET/PTC and those resulting from NTRK1 rearrangements as TRK. Of note, TRK is an extremely rare occurrence. On average, no more than 20% of PTCs have such rearrangements with at least 6 different chimeric RET/PTC and TRK genes shown. TRK may be associated with a more aggressive clinical behavior [16] whilst RET/PTC possibly has a more favorable prognosis [17].The RAF proteins are serine-threonine kinases that activate the RAF/MEK/MAPK signaling pathway. The T1799A mutation of the BRAF gene, occurs in 29%–69% of papillary thyroid cancers [14, 18–20]. The predicted protein product BRAFV600E in PTC is associated with reduced expression of key genes governing iodine metabolism, specifically the impairment of both NIS expression and targeting to membrane leading to dedifferentiation [21, 22]. BRAF mutations have also been shown to be associated with extrathyroidal invasion, lymph node metastasis, and advanced tumor stage hence conferring a worse clinical prognosis compared to BRAF wild-type [23]. Finally, Ras has also been reported in follicular variant of PTC in which no clear evidence of aggressiveness has been found [24]. It is worthy to note that the genetic changes linked with pathogenesis of PTC are mutually exclusive.

The translocation t(2;3)(q13;p25) has been identified in FTC which results in fusion of part of the DNA-binding segment of the PAX8 gene and the peroxisome proliferator-activated receptor gamma 1 (PPAR-gamma-1) gene; PAX8 is a thyroid transcription factor, and PPAR-gamma-1 is a transcription factor that stimulates cell differentiation and inhibits cell growth. The product of the fusion gene blocks the action of the PPAR-gamma-1, an effect that might inhibit cell differentiation and stimulate cell growth [25]. Overexpression of normal c-myc and c-fos genes, as well as mutations of H-ras, N-ras, and K-ras proto-oncogenes, is found in follicular cancers. These abnormalities may confer a growth-promoting effect that may act additively with other oncogene or tumor suppressor gene mutations [26, 27].

The above discovery over the last 5–10 years allowed clinical trials of new therapies for well-differentiated thyroid cancer.

Motesanib diphosphate is an oral tyrosine kinase inhibitor (TKI) targeting the VEGFR 1-3, PDGFR, and cKIT. A multicentre, open-label phase 2 trial was initiated testing the efficacy of this drug in a cohort of 93 patients with progressive DTC. Of the 93 patients, 30% were still on drug after 48 weeks. The partial response (PR) rate was 14% with another 35% maintaining stable disease for at least 24 weeks. The median progression-free interval was 40 weeks. The most common side effects included fatigue, nausea, diarrhea, hypertension, and unexpectedly increased requirements in thyroxine replacement [28].

Axitinib is another oral TKI that blocks VEGFR, PDGFR and cKIT. A multicentre phase II trial examined the efficacy of axitinib in advanced or metastatic thyroid carcinoma of which 75% had DTC. There was 31% PR rate among the DTC patients. Median PFS was 18 months. Common side effects were all typical of this class of drug [29].

Sorafenib is an oral TKI targeting VEGFR 2 and 3, RET and BRAF, cKIT, PDGFR, and FLt-3. Two phase II trials were performed in patients with metastatic PTC. Kloos et al. [30] investigated the use of sorafenib among 36 evaluable patients, and PR was seen in 8%, with a minor response (defined as 23%–29% reduction in tumor diameter) in another 19%. Gupta-Abramson et al. [31] showed that out of 30 patients with metastatic iodine refractory thyroid carcinoma who received sorafenib 400 m twice a day, 23% had a PR lasting up to 84 weeks and 53% achieving stable disease (SD). The median PFS was 79 weeks. In the latest update in abstract form [32], out of 50 evaluable patients, 36% achieved PR and 46% SD with clinical benefit rate of 82%. PFS measured was 84 weeks. The median OS of the initial cohort of 30 patients was 140 weeks. This compares favorably with the historic OS of patients treated with doxorubicin. Patients who harbor BRAFV600E mutation which was a poor prognostic marker in the prekinase era trended towards better outcome when treated with sorafenib compared with patients with wild-type BRAF. Sorafenib is now recognized as a potential therapeutic agent in the NCCN guidelines and is FDA approved for treatment of advanced hepatocellular carcinoma and advanced renal cell carcinoma.

Sunitinib an oral small molecule TKI which demonstrates VEGFRs, RET, and RET/PTC subtypes 1 and 3 inhibition was tested in 31 patients. 13% of the patients achieved PR whilst 68% had documented disease stabilization [33]. Severe adverse effects were similar to that of sorafenib. In another phase II trial where sunitinib was administered on a continuous basis at 37.5 mg every day to 33 patients (26 had well-differentiated thyroid carcinoma), complete response was seen in 2 patients (7%), 25% had PR, and 48% had SD with a clinical benefit rate of 83% [34]. Thus, sunitinib is also available for use in selected thyroid cancer patients with metastatic disease outside of clinical trial. Despite early promise of gefitinib demonstrating efficacy in preclinical models on the premise of many papillary thyroid cancers displaying activated EGF receptor signaling, the phase II study was met with disappointing results. There was no documented objective response with median progression of only 4 months [35].

Two phase 2 studies have just been reported in ASCO 2010 in refractory thyroid carcinoma. AZD6244, an oral inhibitor of mitogen-activated protein kinase, MEK-1/2, was found to result in median progression-free of 32 weeks [36]. Aflibercept, a VEGF-trap, was evaluated in 21 patients culminating in stable disease rate of 83% [37].

In a phase II trial, 29 patients with metastatic differentiated thyroid cancer were treated with increasing doses of thalidomide. PR was reported in about 20 percent of patients, SD was seen in about 35 percent, and there were no complete responses. Most common and serious toxicities included fatigue, infection, and neuropathy, and half of patients discontinued therapy due to side effects [38]. Twenty one patients were treated with 25 mg on lenalidomide in an open label phase two trial with PR of 22% and SD 44%. Treatment was well tolerated with mainly grade 3 hematological toxicities [39].

There are clearly multiple agents which have been tested at phase II levels which have tremendous results warranting validation in phase 3 clinical trials. Future trials should perhaps investigate efficacy of combination targeted therapy with chemotherapy in first line treatment of advanced thyroid cancer. There are currently a few phase I/II trials underway investigating other targets such as BRAFV600E selective kinase inhibitor, proteaosome inhibitor, and PPAR-gamma agonist. Everolimus, an mTOR inhibitor, has shown preclinical activity and warrants further testing [40].

## 3. Anaplastic Thyroid Carcinoma (ATC)

ATC is uncommon and comprises 1%-2% of thyroid malignancies. ATC typically affects elderly patients with median age of 68 at diagnosis. It has a fulminant course with almost 100% disease-specific mortality. The median survival is approximately 4 months with one year overall survival of 16%–19% [41].

ATC may originate de novo or from preexisting PTC or FTC. A number of gene mutations have been identified. Mutation of the p53 tumor suppressor gene has been identified in up to 70% of anaplastic thyroid cancers [42]. Production of inactive p53 protein leads to dysregulation of both apoptosis and the cell cycle. Mutations within the *β*-catenin gene occur in up to 65% of ATC [43]. The beta catenin protein plays a role in cell adhesion and in signaling through the Wnt pathway. The exon 3 mutation is associated with increased nuclear localization of the beta catenin resulting in increased nuclear signaling and hence tumorigenesis or tumor progression.

It seems likely that in some PTC, dedifferentiation may lead to ATC. This is highlighted by studies showing between 15%–50% of ATC containing BRAF mutation [44, 45]. Interestingly, the incidence of BRAF mutation has been reported to be as high as 62% in radioactive iodine-refractory thyroid cancers [46]. Mutations involving RAS (regulates the RAS-RAF-MEK-ERK and PI3K/AKT1 pathways) are found to affect between 6%–50% of ATC [47, 48]. Somatic mutations of the catalytic subunit of the phosphatidylinositol 3′-kinase (PIK3CA) have been identified in about 23 percent of anaplastic thyroid cancers [49].

Clinical management of anaplastic thyroid cancer hinges on retrospective series of major centres owing to its rarity and thus lack of randomized studies.

For ATC localized to the thyroid, complete resection should be attempted in conjunction with postoperative adjuvant therapy. At Roswell Cancer Institute, the 5-year overall survival of the patients who achieved complete resection was 60% [50]. In contrast, Kobayashi et al. [51] reported a 1-year survival rate of 20% for patients who had complete resection whilst no one with residual disease was alive. Haigh et al. [52] managed 33 patients and found surgical resection with curative intent in eight which resulted in median survival of 43 months and 5-year survival of 50%. Most received adjuvant chemoradiotherapy. Meanwhile, De Crevoisier [53] reported high long-term survival when radio-chemotherapy was administered after complete resection. These patients received 6 cycles of doxorubicin (60 m/m²) and cisplatin (120 ng/m²) with 40 Gy of external beam radiotherapy (EBRT). At the end of treatment, complete local response was seen in 19 patients. With a median followup of 45 months, 7 patients were still in complete remission of which 6 had previously achieved complete resection. All the above together with SEER analysis lends support to adjuvant radio/chemotherapy in improving survival outcomes. However this is confounded by the fact that patients who received adjuvant treatment usually have less extensive disease.

There is no effective therapy for advanced or metastatic anaplastic thyroid cancer, and the disease is uniformly fatal. The median survival from diagnosis ranges from three to seven months, and the one-year survival rate ranges 20%–35% [50, 52].

ECOG conducted a prospective trial from 1976 to 1982 whereby 21 patients were randomized to doxorubicin versus 18 randomized to doxorubicin plus cisplatin. The single agent arm had one partial response while the 2-drug regimen produced 3 complete responses and 3 partial responses. Median survival however was only 2.7 months. There were however 2 prolonged CRs lasting 35 and 41 months [54]. Ain et al. conducted a phase II clinical trial exploring efficacy of paclitaxel as a 96-hour infusion. The objective response rate was 52%. Overall survival was 6 months with a higher median survival of 32 weeks in the 10 responders [55].

Like its differentiated counterpart, understanding of the molecular pathogenesis has allowed studies on novel targeted agents to be carried out. Combretastatin A-4 phosphate (CA4P), a tubulin binding disrupting agent, showed some interesting findings in the study of Dowlati et al. [56]. One of the 3 patients with ATC achieved complete response and was alive at 30 months. However, in a later phase II trial, out of 18 patients, no objective responses were seen; 6 had stable disease, and 12 patients progressed with median survival of 20 weeks. It appears that CA4P has limited efficacy as a single agent, and perhaps combination with either chemotherapy or another targeted agent may be beneficial [57]. Combination of high-dose intermittent gefitinib was evaluated in a phase I trial, and one patient with ATC had a 4-month partial remission [58]. Cohen et al. evaluated axitinib in phase II trial. Two patients with ATC were enrolled. One had a PR of 4 months whilst the other patients progressed [29].

Several agents are currently being tested in phase I or II settings for advanced anaplastic thyroid cancer including CS7017, an oral PPAR inhibitor, in combination with paclitaxel, imatinib and bavituximab in combination with doxorubicin and pemetrexed with paclitaxel.

## 4. Medullary Thyroid Carcinoma (MTC)

Medullary thyroid cancers (MTCs) are tumors of thyroid parafollicular C cells which are part of the amine precursor uptake decarboxylation (APUD) system rather than thyroid epithelial cells. MTCs do not concentrate iodine. They may occur as sporadic tumors, as a component of multiple endocrine neoplasia type (MEN) 2, or as a familial MTC syndrome without MEN association. The gender distribution is equal. The tumor is unilateral in majority of sporadic forms but mostly bilateral and multifocal in familial cases. They secrete calcitonin and sometimes carcinoembryonic antigen (CEA), both of which can serve as tumor markers [59]. Calcitonin production is stimulated by both calcium and pentagastrin. This forms the basis of the stimulation tests using calcium infusion [60, 61]. The primary treatment for MTC is extensive and meticulous surgical resection. Total thyroidectomy is frequently needed in addition to complete resection of lymph nodes in the central neck, paratracheal and upper mediastinal region. Fortunately, mutations in the RET proto-oncogene have now been established in MEN-2. Family members can be screened at birth, obviating yearly provocative testing and allowing affected members to be offered early thyroidectomy [62].

Among patients with recurrent local or distant disease, the rate of progression is variable, and some patients survive for years despite obvious metastatic disease. This fact also makes assessment of the efficacy of any therapy very difficult; about all that can be done is to determine whether a therapy leads to tumor shrinkage. Despite these limitations, it seems clear that external beam radiotherapy is rarely effective, and chemotherapy has only occasionally been effective.

The treatment of MTC using cytotoxic drugs has mirrored the historical development of chemotherapy use in neuroendocrine tumors. Single agent regimens have been characterized by low response rates (<10%) and/or lack of durable response. Doxorubicin, aclarubicin, mitoxantrone, and streptozocin were the agents investigated previously as monotherapy [63–65]. Dacarbazine in combination with other drugs is recommended for patients with metastatic MTC in guidelines from the National Comprehensive Cancer Network [66]. Dacarbazine plus 5-fluorouracil (5-FU) is an active combination. In one series, three of five patients had a partial response, while in a second study, one patient had a complete remission lasting ten months. In preclinical studies, the novel tubulin-binding agent combretastatin A-4 phosphate, in combination with doxorubicin, was active in a xenograft model of MTC in nude mice. Preclinical evidence from the same centre also suggested activity for irinotecan in MTC cell lines [67].

Other modalities evaluated including interferon alpha, long-acting somatostatin analog, yttrium-90-labelled somatostatin analogs, and immunotherapy have largely found to confer marginal clinical benefit [68–74].

A marine cyclodepsipeptide, aplidin (plitidepsin), extracted from a Mediterranean tunicate was investigated in a phase I trial involving six patients with MTC out of 67 with thyroid cancers. Four of the six had stable disease for over six months [75]. Since many MTC patients have spontaneous disease stabilization, this is not definitive results; however this drug merits further investigation. A small molecule inhibitor, arylidene-2-indolinone (RPI-1), abolishes the constitutive tyrosine phosphorylation caused by the specific RET proto-oncogene mutation seen at cysteine residue 634 in the syndrome multiple endocrine neoplasia type 2A (MEN2A). Hopefully, this agent will have future applications both in inherited MTC as well as in papillary cancers associated with the RET/PTC1 gene rearrangement [76].

Another novel therapeutic approach includes modulation of angiogenesis considering that VEGF is elevated in 75% of metastatic MTC [77]. Several phase 2 studies have been conducted to evaluate the efficacy of this strategy. 91 patients with advanced/metastatic medullary thyroid cancer were treated with motesanib diphosphate in a multicentre trial. Although the objective response rate was only 2%, almost half of the patients experienced prolonged stable disease [78]. Similarly, sunitinib was noted to confer an overall response rate of 35% and clinical benefit rate of 91% in another study involving 25 patients [79]. Building on the knowledge that germline or somatic mutations of RET account for 75% of MTC, a randomized, double-blind phase 3 trial involving 331 patients was conducted utilizing vandetanib an oral inhibitor of RET, VEGFR, and EGFR. Although the overall survival analysis is immature it is assuring to note that patients treated with vandetanib had a significantly longer progression-free survival compared to the placebo group (HR 0.45, *P* = .0001) [80]. Antitumor activity has been noted in XL184, an oral inhibitor of RET, MET, and VEGFR2. Hepatocyte growth factor (FGF) receptor tyrosine kinase (MET) has also been detected in MTC and transduction of normal human thyroid cells with mutant RET results in upregulation of MET. Moreover preclinical model suggests that MET and VEGFR2 play synergistic roles in promoting tumor angiogenesis and dissemination [81]. A phase 1 dose finding study involving 37 patients with MTC yielded PR rate of 29% and prolonged SD of 68% [82]. The efficacy of XL 184 is currently being evaluated in a randomized phase 3 trial.

## 5. Conclusion

The improved understanding of the molecular basis of pathogenesis and development of thyroid cancers of follicular epithelial origin and medullary thyroid cancers has vastly increased the armamentarium of therapeutic options available to the patient with metastatic disease. A continued rational approach to the development of these therapeutic modalities will further define their future clinical applications.
